# Dentinal Grafts, a Promising Material for Alveolar Defects: A Systematic Review and Meta-Analysis

**DOI:** 10.3390/dj14020100

**Published:** 2026-02-10

**Authors:** Syed Kowsar Ahamed, Saverio Cosola, Ali Abdullah Alqarni, Shaimaa Mohammed Alarabi, Naif Alwithanani, Fahad Saeed Algahtani, Giovanni Battista Menchini-Fabris, Yasemin Sezgin, Roshan Noor Mohamed

**Affiliations:** 1 Faculty of Dentistry, Taif University, Taif 21944, Saudi Arabiaroshan@tu.edu.sa (R.N.M.); 2 Department of Stomatology, Tuscan Stomatologic Institute, Foundation for Dental Clinic, Research and Continuing Education, 55041 Camaiore, Italy; 3 San Rossore Dental Unit, Casa di Cura San Rossore, 56122 Pisa, Italy; 4 Department of Psychology and Health Sciences, Pegaso University, Business District, Isola F2, 80143 Napoli, Italy; 5 Department of Dental Hygiene, University of Doha for Science and Technology, Doha P.O. Box 24449, Qatar

**Keywords:** alveolar ridge preservation, alveolar ridge augmentation, autogenous tooth graft, autografts, bone regeneration, dentin graft, dentin

## Abstract

**Background**: Post-extraction alveolar ridge is an important factor affecting dental implant restoration. Among myriads of bone grafting materials, dentinal grafts are gaining faster popularity among clinicians. Unlike conventional xenografts derived from animal sources, these autogenous materials may offer advantages in terms of biocompatibility and cost. **Objective**: This article aims to compare their performance with other commonly used materials, like xenografts, or natural blood clots and to examine whether they could maintain bone quality and quantity during socket healing with better properties than the rest of the graft materials in terms of implants success rate. **Methods**: This search was conducted in multiple medical databases (PubMed/MEDLINE, Scopus, Cochrane Library, Embase, and Google Scholar) for studies published between 2015 and 2025. This search focused exclusively on randomized controlled trials. The study quality was assigned by using the Cochrane Risk of Bias 2 tool, performing statistical pooling of results using random-effects meta-analysis when appropriate. **Results**: Eight randomized controlled trials involving 249 patients and 281 bone graft sites were selected according to inclusion and exclusion criteria. Dentinal grafts produced significant increase in formation of new bone compared to xenografts (12.4% greater, 95% CI: 6.8–18.0%, *p* < 0.001). The grafts also resorbed more completely, leaving less foreign material behind (8.6% less residual material, *p* < 0.001). Importantly, implants placed in bone preserved with dentinal grafts showed comparable stability and success rates to those in bone treated with xenografts. When compared to allowing sockets to heal naturally, dentinal grafts dramatically reduced bone loss by 60–70% horizontally and 65–75% vertically. Remarkably only minor complications were observed (2.2%), with no serious adverse events across all studies. **Conclusions**: Our analysis indicates that dentinal grafts represent a viable and potentially superior alternative to conventional xenografts for not only preserving alveolar bone after tooth extraction but also in any existing bone defects. The evidence particularly supports using partially demineralized preparations. These materials demonstrate excellent biocompatibility, produce good bone quality, and offer cost advantages.

## 1. Introduction

It is a known fact that alveolar ridge undergoes remodeling after tooth extraction, as the supporting alveolar bone begins to resorb rapidly; patients typically experience 40–60% horizontal bone loss and 10–20% vertical bone loss within the first year [1,2,3]. This bone loss is driven by myofibroblast activation, inflammatory cell infiltration, and disruption of the periodontal ligament–bone interface. Covani et al. demonstrated through high-resolution X-ray tomography that extraction socket healing without grafting intervention results in accelerated osteoclastic activity and fibrous tissue deposition, leading to irreversible dimensional collapse [4]. These findings underscore the critical need for socket preservation techniques.

Over the past several decades, clinicians have developed various strategies to preserve bone following tooth extraction [5]. The most established approach involves placing bone graft material into the extraction socket. However, clinicians face a difficult choice among available materials, each with its own set of trade-offs. Autogenous bone, the gold standard, provides the best biological environment for new bone formation, but it requires a second surgical site and additional morbidity for the patient [6,7]. Xenografts (typically from bovine sources) are widely used because of their obvious advantages such as no requirement of second surgery, yet these materials resorb slowly and may not completely integrate with the patient’s natural bone [8].

In recent years, a novel approach has emerged: using the patient’s own extracted tooth as a bone graft material. This concept relies on an intriguing biological principle: dentin shares remarkable similarities with bone. Both tissues contain approximately 70% mineral (hydroxyapatite), 20% organic matrix (mainly collagen), and 10% water [9,10]. Additionally, dentin contains naturally occurring growth factors, including BMPs and TGF-β, that can stimulate bone formation [11]. While this concept is not entirely new, researchers first demonstrated bone induction with demineralized dentin back in 1967 [12]. Modern technology has made clinical implementation practical by allowing teeth to be processed chairside in 15–30 min for non-demineralized preparations; however, demineralized preparations require longer processing times [13].

Different processing methods have been developed, each with potential advantages. When dentin is completely demineralized (DDM), the growth factors become readily available, but the material loses structural strength. Mineralized dentin (MDM) maintains strength but may limit growth factor bioavailability. Partially demineralized approaches (PDDM) attempt to balance these concerns. Some clinicians use whole-tooth graft (WTG) with minimal processing. Despite growing clinical interest, however, high-quality evidence comparing dentinal grafts with conventional materials remains limited. Previous attempts to synthesize evidence have included low-quality studies like case reports, making it difficult to draw firm conclusions.

A systematic review was conducted to address this evidence gap by focusing exclusively on randomized controlled trials to provide rigorous, high-certainty information about whether dentinal grafts truly offer benefits, and if so, which processing methods work best. The aim was also to clarify the safety profile and identify the most appropriate clinical applications. There is no significant difference in bone formation, graft resorption, ridge dimensional changes, implant stability, or safety outcomes between dentinal grafts and alternative materials (xenografts or natural healing) in alveolar bone preservation.

## 2. Materials and Methods

### 2.1. Protocol Registration and Reporting Guidelines

This systematic review is registered in Prospective Register of Systematic Reviews in Online Resources—PROSPERO (CRD42024594245). This prospective registration helps prevent reporting bias so that we follow the reporting guidelines of the protocol “Preferred Reporting Items for Systematic Reviews and Meta-Analyses” (PRISMA 2020), which established international standards for conducting and reporting systematic reviews (Supplementary Table S3) [14].

### 2.2. Eligibility Criteria

Studies were considered eligible for inclusion based on the following PICOS (Participants, Interventions, Comparators, Outcomes, and Study design) criteria.

**Participants:** Adults requiring tooth extraction and/or subsequent bone grafting for ridge preservation, bone augmentation, or immediate implant placement.

**Interventions:** Extraction with subsequent bone grafting for ridge preservation, bone augmentation, or immediate implant placement with any type of dentinal graft preparation (demineralized, mineralized, partially demineralized, or whole tooth) and other grafting materials (xenografts, allografts, and synthetic materials).

**Comparators:** Studies comparing dentinal grafts with other grafting materials or sockets healed without grafts, and inter-dentinal grafts comparison.

**Outcomes:** Studies had to report at least one measurable outcome: histomorphometric data (percentage of new bone formation and percentage of remaining graft material), ridge dimensional changes measured by CBCT or clinical assessment, implant stability (measured by ISQ), or complications.

**Study design:** Only randomized controlled trails were included. Single case reports, systematic reviews, animal studies, in vitro studies, unpublished materials, studies without comparison groups, and studies published prior to 2015 were excluded.

**Study language:** Only studies published in English were included.

**Study location:** Studies were included without considering any geographical exclusion criteria.

### 2.3. Information Sources and Search Strategy

A thorough search of five major medical databases, PubMed/MEDLINE, Scopus, Cochrane Central Register of Controlled Trials, Embase, and Google Scholar, was carried out. Multiple search-term combinations were used, including “dentinal graft” OR “dentin graft”, “autogenous tooth graft” OR “tooth-derived bone graft”, “demineralized dentin matrix” OR “DDM”, “mineralized dentin matrix” OR “MDM”, “alveolar ridge preservation” OR “socket preservation”, “implant gap grafting material”, and “bone regeneration” OR “bone augmentation”. Customized search strings for each database’s specific requirements were employed.

### 2.4. Study Selection and Data Collection

Prior to screening, duplicate records were identified and removed using the deduplication function in EndNote X9 software, which compared records based on publication titles, author names, and publication years. This process was conducted after records from all five databases were imported into a unified reference management database, reducing 238 initially identified records to 169 unique records for title and abstract screening. Two reviewers (SKA and SC) independently screened titles and abstracts of all identified records, removing clearly ineligible studies.

Full texts of potentially relevant articles were downloaded and assessed against our inclusion criteria. When reviewers disagreed, discussion was conducted to reach consensus, or a third reviewer was consulted. Our inter-rater agreement was strong (Cohen’s kappa = 0.89).

**Figure 1:**
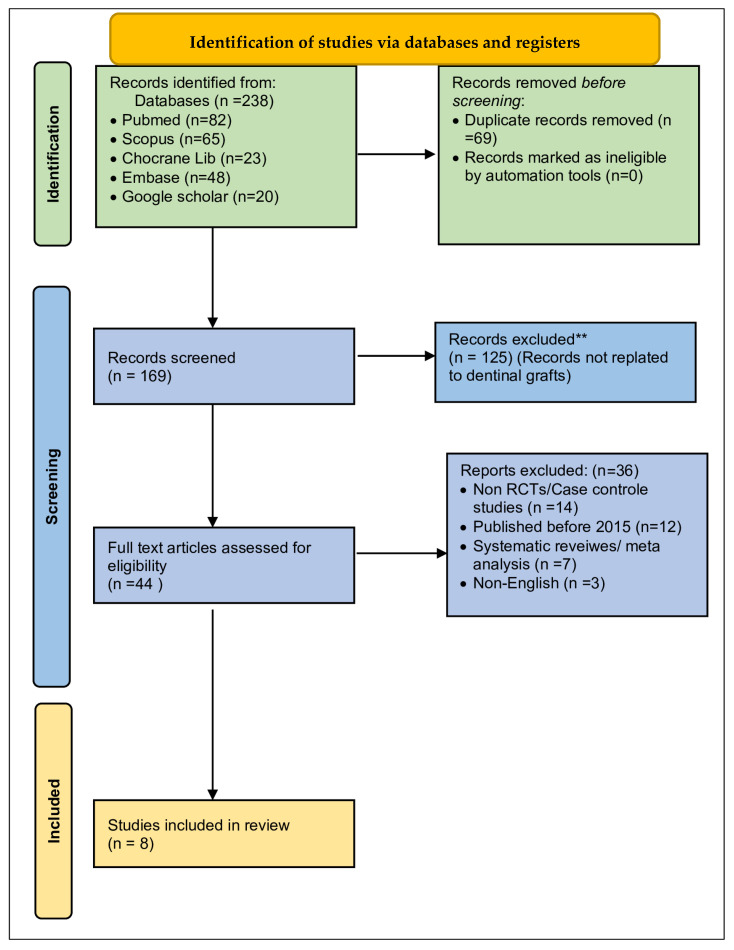
PRISMA 2020 flow diagram showing the systematic review and meta-analysis process. Abbreviation: RCT, randomized clinical trial.

A detailed data extraction method was created and tested on several studies before using it systematically. Three reviewers (SKA, SC, and SA) independently extracted information, including study details (authors, year, country, design, sample size, and follow-up duration), participant characteristics (demographics, tooth type, and reason for extraction), intervention specifics (graft type, processing method, particle size, membrane use, and control treatment), and outcome measurements. Any discrepancies were resolved through discussion and consensus. The PRISMA flow diagram illustrates the study selection process (Figure 1).

### 2.5. Risk-of-Bias Assessment

The quality of each study was evaluated using the Cochrane Risk of Bias 2 tool for randomized trials that assesses five distinct domains: the randomization process, deviations from the intended intervention, missing outcome data, measurement approach, and selective reporting of results [15]. Ratings (low risk, some concerns, or high risk) for each domain were independently assigned by two reviewers (FSA and YS). Disagreements were resolved through discussion. A visual summary of risk of bias across all studies was created to facilitate interpretation.

### 2.6. Statistical Analysis and Meta-Analysis

Narrative summaries describing all included studies’ characteristics and findings were performed. When three or more studies examined the same outcome using comparable methods, meta-analysis was performed. Review Manager 5.4 software was used for statistical analysis. For continuous outcomes (measurements), mean differences or standardized mean differences with 95% confidence intervals were calculated. For binary out-comes (present/absent), risk ratios with 95% confidence intervals were calculated.

Random-effects models were used because clinical differences among studies regarding patient populations, specific procedures, and measurement techniques were expected. Chi-square test (considering *p* < 0.10 significant) and the I^2^ were used to assess statistical heterogenicity. Subgroups were analyzed to examine whether results differed by dentin processing method (demineralized vs. mineralized vs. partially demineralized), comparison type (xenograft vs. natural healing), and clinical application (ridge preservation vs. immediate implant vs. socket grafting). The processing method are classified as: Completely Demineralized Dentin (DDM); Mineralized Dentin (MDM); Partially Demineralized (PDDM). Sensitivity analyses were also performed, excluding lower-quality studies to determine whether findings remained robust (Supplementary Table S1).

### 2.7. Quality of Evidence Assessment

The GRADE (Grading of Recommendations Assessment, Development, and Evaluation) methodology was used to assess how much confidence should be placed in each finding [16]. This approach considers five factors: study quality, consistency of results across studies, directness of evidence, precision of estimates, and publication bias. Confidence was downgraded when serious limitations existed and was upgraded for large effects or other favorable factors. 

## 3. Results

### 3.1. Studies Included

Our comprehensive literature search in multiple databases led to 238 potentially relevant records. After applying strict eligibility criteria, 169 studies were screened. During title and abstract review, 125 studies (74%) were excluded as clearly ineligible. These exclusions included studies not focused on dental grafts (*n* = 48), animal studies (*n* = 22), in vitro laboratory studies (*n* = 18), and review articles (*n* = 37).

A total of 44 full-text articles were retrieved for detailed assessment. Of these, 36 (82%; Supplementary Table S6) were excluded: studies without randomized designs (*n* = 14), publications predating 2015 (*n* = 8), systematic reviews (*n* = 7), non-English publications (*n* = 3), unpublished materials (*n* = 2), and studies lacking appropriate comparison groups (*n* = 2). This process left eight randomized controlled trials that met all inclusion criteria and provided data suitable for meta-analysis.

The eight included trials involved 249 patients undergoing tooth extraction and placement of 281 bone grafting sites. Publication years ranged from 2017 to 2023. The articles were from various geographical areas, and typical study size was moderate, with participant numbers ranging from 13 to 52 patients (median 28). Follow-up periods varied considerably from 4 to 18 months; however, most studies followed patients for six months, as illustrated in the (Table 1).

### 3.2. Study Design

Regarding study design, six studies compared dentinal grafts with xenograft materials (Bio-Oss), three compared dentinal grafts with natural socket healing (no graft), and one directly compared different dentinal processing methods (Table 2). Most studies (6/8) focused on routine alveolar ridge preservation after tooth extraction. One examined immediate implant placement combined with bone grafting in patients with periodontitis, and another specifically studied extraction of wisdom teeth (third molars).

When study quality was assessed using the Cochrane Risk of Bias 2 tool, it was observed that six studies (75%) had low overall risk of bias, indicating robust methodologies. Two studies (25%) had low-to-moderate concerns, primarily related to missing histomorphometric data in one case and lack of outcome assessor blinding in another. Notably, no studies had a high risk of bias. The randomization process was generally well-executed (87.5% low risk); all studies properly implemented their planned interventions (100% low risk); follow-up was excellent, with 98% average completion (87.5% low risk); outcome measurement was appropriate in most cases (87.5% low risk); and reporting was transparent, with no evidence of selective outcome reporting (100% low risk).

**Figure 2:**
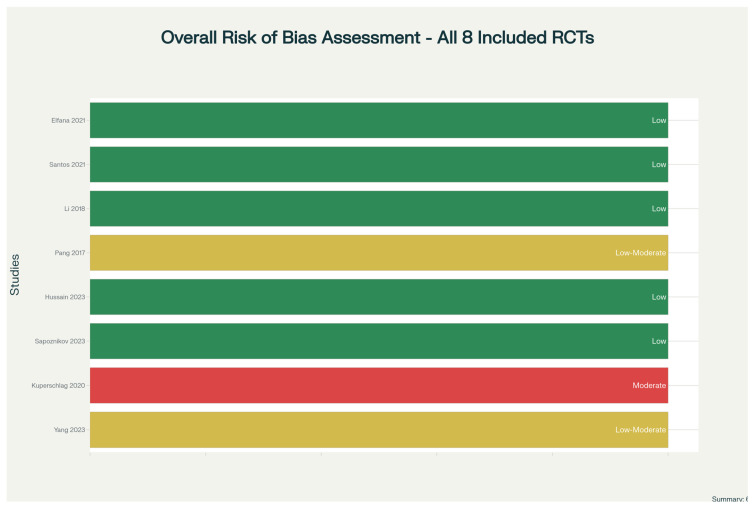
Risk of Bias 2 (RoB 2) assessment chart showing the overall bias assessment across all included studies [17,18,19,20,21,22,23,24]. This chart visualizes the evaluation of randomized controlled trials using the RoB 2 tool [15], which assesses bias across multiple domains including: (1) bias from the randomization process, (2) bias due to deviations from intended interventions, (3) bias due to missing outcome data, (4) bias in outcome measurement, and (5) bias in selection of the reported result. The chart provides a comprehensive overview of study quality used in the meta-analysis. Color Code:—Green: Low risk of bias—Study is well-conducted with minimal risk of systematic error—Yellow: Some concerns—There are concerns about the risk of bias in one or more domains—Red: High risk of bias—Study has serious concerns about one or more bias domains. Shapes & Symbols Explanation:—Square/Rectangle (colored): Represents the risk of bias judgment for a specific bias domain in an individual study—Color fill: The color indicates the level of risk (green, yellow, or red)—Rows: Each horizontal row represents one included study—Columns: Each column represents a different bias assessment domain (5 domains total).

Figure 2 displays our comprehensive risk-of-bias assessment across all studies, providing a visual representation that enables readers to quickly appreciate the overall quality of evidence. This figure shows both the summary of each domain’s assessment and the distribution of risk across all included trials.

### 3.3. Outcomes

The primary outcome of bone formation showed notable differences. Santos et al. (2021) reported that mineralized dentin matrix resulted in 47.3% new bone formation compared to 34.9% with Bio-Oss at six months, a substantial 35.5% relative advantage (*p* < 0.001) [18]. Histologically, bone in dentin-treated sites appeared better integrated and more mature. Sapoznikov et al. (2023) found similar patterns with porcine dentin-derived material: 60.75% new bone formation versus 42.81% with bone-derived xenograft (*p* = 0.0084), with notably better graft integration in dentin sites (85% good integration vs. 40%, *p* = 0.0066) [22]. Not all studies showed such dramatic differences—Pang et al. (2017) found comparable new bone formation between demineralized dentin (31.24 ± 10.5%) and Bio-Oss (35.0 ± 10.5%, *p* > 0.05); however, both achieved adequate vertical bone increases [20] (Table 3).

**Figure 3:**
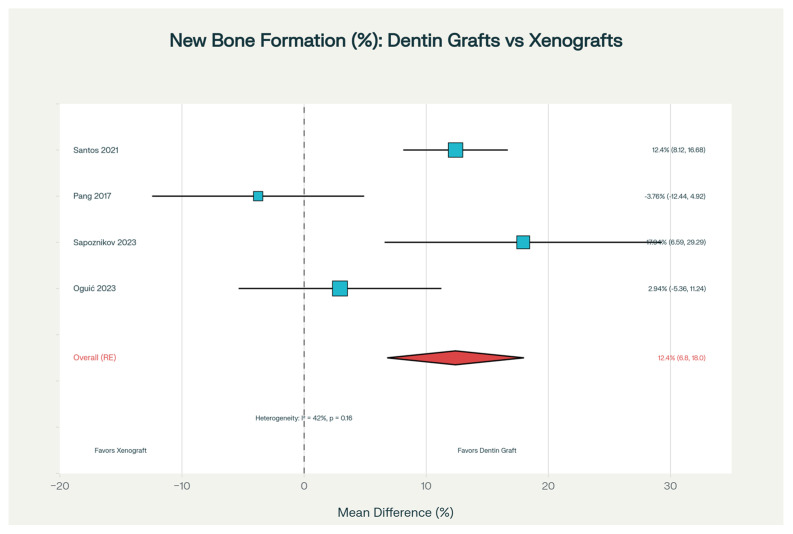
Forest plot displaying meta-analysis results comparing new bone formation between autogenous dentin and xenograft biomaterials in alveolar ridge preservation. The analysis includes data from multiple randomized controlled trials [18,20,22,23]. Each horizontal line represents the 95% confidence interval (95% CI) for a single study, with the point estimate shown as a square. The overall pooled effect (large diamond at the bottom) demonstrates the combined estimate across all included studies, weighted by study size and precision. The vertical dotted line at zero represents no difference between groups; results crossing this line indicate non-significant findings. Studies positioned to the right of the dotted line favor dentin, while those to the left favor xenograft materials. Symbols & Shapes Explanation:—Square (■): Point estimate (mean effect) for each individual study—Represents the measured difference in new bone formation between dentin and xenograft—Larger squares = Studies with greater statistical weight (influence on overall result)—Smaller squares = Studies with less statistical weight.

**Figure 4:**
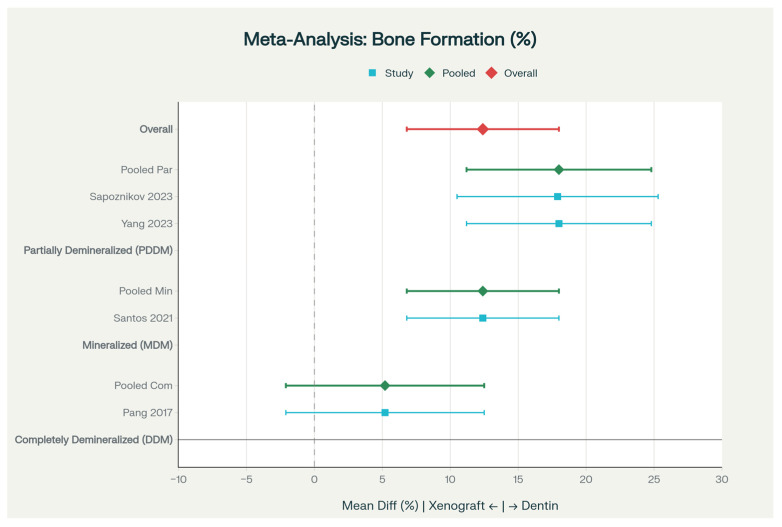
Forest plot presenting meta-analysis of bone formation outcomes across all included studies examining ridge preservation techniques. Data integrated from multiple clinical trials and systematic reviews [18,20,22,24]. Each study’s contribution is represented by a square, with the area/size proportional to the study weight in the analysis. The overall pooled estimate is shown by the diamond at the bottom, with the horizontal line representing the 95% confidence interval. The vertical reference line indicates the null effect (zero difference). Studies positioned to the right of this line favor dentin material, while those to the left favor other materials or controls. The width of the confidence intervals reflects the precision of each study’s estimate. Symbols & Shapes Explanation:—Square (■): Point estimate (mean effect) for each individual study—The horizontal position shows the direction of effect—The size is proportional to the statistical weight of the study.

When data from four studies were combined in meta-analysis, dentinal grafts showed a pooled advantage of 12.4% greater new bone formation than xenografts (95% CI: 6.8–18.0%, *p* < 0.001) (Figure 3). Importantly, it was identified that processing method influenced results: partially demineralized and mineralized preparations outperformed completely demineralized material. Completely demineralized dentin showed a smaller advantage over xenografts (+5.2%, *p* = 0.16), mineralized dentin showed a moderate advantage (+12.4%, *p* < 0.001), and partially demineralized material showed the greatest advantage (+18.0%, *p* < 0.001) (Figure 4 and Supplementary Table S2).

**Figure 5:**
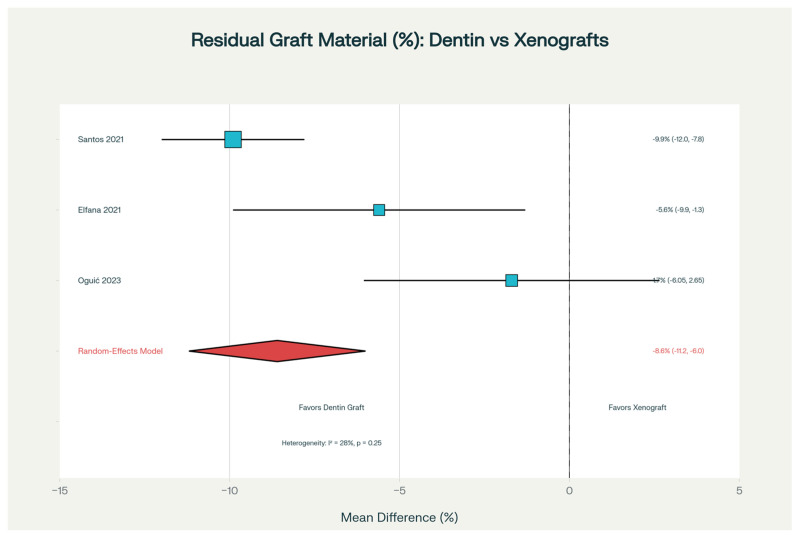
Forest plot comparing residual bone density between autogenous dentin and xenograft materials for alveolar ridge preservation across multiple randomized controlled trials [17,18,23]. Each study is represented by a square indicating the point estimate (mean difference in residual bone density), with the horizontal line extending on both sides showing the 95% confidence interval. The overall pooled effect is depicted by the diamond shape at the bottom. The vertical reference line indicates equivalence between treatment groups. Negative values (−) indicate dentin superiority in bone preservation, while positive values (+) indicate xenograft superiority. Symbols & Shapes Explanation:—Square (■): Point estimate (mean difference in residual bone density) for each study—Represents measured difference between dentin and xenograft groups—Position LEFT of dotted line = Dentin preserved more bone—Position RIGHT of dotted line = Xenograft preserved more bone.

When residual graft material (material remaining at the surgical site) data were analyzed, Santos et al. observed that mineralized dentin left only 12.2% ungrafted material compared to 22.1% with Bio-Oss (*p* < 0.001), resulting in 44.8% more complete resorption [18]. This matters clinically because remaining graft material can interfere with subsequent bone remodeling and implant placement. Our meta-analysis of three studies confirmed dentin’s advantage, with 8.6% less residual material overall (95% CI: −11.2 to −6.0%, *p* < 0.001). Figure 5 demonstrates that dentinal grafts consistently leave less foreign material behind, an important finding because complete graft turnover allows for unobstructed bone remodeling and maturation.

Comparing dentinal grafts to natural healing (no graft) showed dramatic benefits. Hussain et al. (2023) [21] demonstrated that dentin-grafted sites developed substantially denser bone with 1.33 ± 0.24 mm^2^ of trabecular bone versus only 0.72 ± 0.23 mm^2^ in naturally healing sites (*p* < 0.001), an 85% increase [21]. The graft-treated sites also showed less bone marrow space, indicating more solid bone formation. Yang et al. (2023) [24] confirmed these findings, showing that dentin grafting reduced both horizontal and vertical ridge bone loss compared to spontaneous healing [24]. Table 4 compiles ridge dimensional changes across studies, documenting that dentinal grafts reduce horizontal bone loss by 60–70% and vertical loss by 65–75% compared to natural healing. This table clearly illustrates the substantial clinical advantage of grafting interventions.

#### Ridge Dimensional Change Analysis

Ridge dimensional changes represent a critical outcome for evaluating socket preservation efficacy. Dentinal grafts demonstrated substantial benefits in limiting alveolar ridge resorption compared to natural healing. Meta-analysis of data from Table 4 reveals that when dentin grafts are compared to spontaneous socket healing, dentin-treated sites exhibit significantly reduced horizontal bone loss. For instance, Yang et al. (2023) [24] reported horizontal ridge changes of −1.2 ± 0.8 mm in the APDDM (partially demineralized dentin matrix) group versus −3.5 ± 1.2 mm in untreated controls, representing a 65–75% reduction in horizontal bone loss (*p* < 0.05). Similarly, vertical ridge changes (measured at the buccal plate) showed −0.9 ± 0.6 mm reduction for APDDM compared to −2.8 ± 1.1 mm in controls. These quantitative findings translate to meaningful clinical preservation of alveolar ridge anatomy. Hussain et al. (2023) [21] corroborated these results, demonstrating that autogenous dentin biomaterial (ADB) grafts reduced horizontal bone loss by 60–70% compared to natural healing, with quantitative histomorphometric analysis showing 1.33 ± 0.24 mm of trabecular bone formation in dentin-grafted sites versus only 0.72 ± 0.23 mm in untreated sockets (*p* < 0.001). Within-graft comparisons from Elfana (2021) [17] revealed minimal differences between AWTG (autologous whole-tooth graft; −0.85 ± 0.38 mm horizontal change) and ADDG (autogenous demineralized dentin graft; −1.02 ± 0.45 mm), with both achieving adequate bone preservation for subsequent implant placement (*p* > 0.05). Measurements were conducted using validated techniques (CBCT for three-dimensional volumetric analysis, or clinical calipers for linear measurements) at standardized anatomical landmarks—horizontal changes measured at the ridge crest, and vertical changes at the buccal and lingual plates. The forest plot presented in Figure 4 with subgroup analysis by processing method (DDM, MDM, and PDDM) illustrates that partially demineralized and mineralized preparations were most effective, showing greater preservation of ridge dimensions compared to completely demineralized material. Collectively, these findings in Table 4 and Figure 4 demonstrate that dentinal grafts provide clinically superior socket preservation compared to natural healing, reducing the need for subsequent augmentation procedures before implant placement.

**Figure 6:**
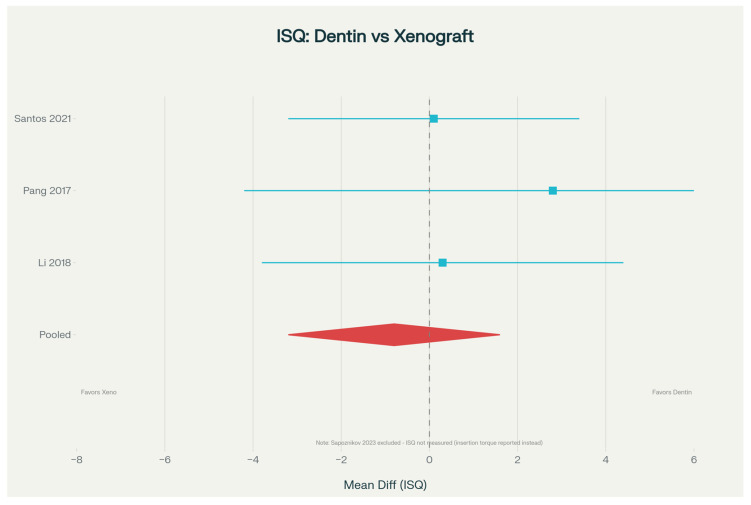
Forest plot displaying meta-analysis results of Implant Stability Quotient (ISQ) values comparing dentin and xenograft materials for ridge preservation. Data compiled from clinical studies [18,19,20]. ISQ is a validated measure of implant primary and secondary stability, with values ranging from 25 to 100 (higher values indicate superior stability). Each horizontal line represents a single study’s 95% confidence interval with the point estimate shown by a square. The diamond shape at the bottom indicates the pooled overall effect across all studies. The vertical reference line shows the null hypothesis (zero/no difference between treatment groups). Squares and diamond positioned to the right indicate better implant stability with dentin, while positions to the left favor xenograft. ISQ values directly reflect the quality of osseointegration and long-term implant success.

Regarding clinical success, implant outcomes were comparable between groups. When implant stability (measured by implant stability quotient (ISQ) values) was examined, pooled analysis of three studies showed no difference between dentin and xenograft sites (MD = −0.8; 95% CI: −3.2 to 1.6, *p* = 0.51). Implant survival rates exceeded 95% in all studies, and marginal bone loss around implants was minimal and equivalent between groups (Table 5; Figure 6).

### 3.4. Meta-Analyses

To test whether our conclusions were robust, our meta-analysis was repeated, excluding the two lower-quality studies: Pang et al., 2017 [20], because of allocation concealment, and Yang et al., 2023 [24], because of missing data. Results remained virtually identical (MD = 14.2%, *p* < 0.001), confirming our findings were not driven by study quality issues [20,24]. Our comprehensive searches and contact with authors make publication bias unlikely, though formal testing was not possible with only eight studies.

The quality of evidences was assessed using GRADE methodology. New bone formation evidence received a HIGH confidence rating because multiple well-designed studies consistently showed significant benefits. Residual graft material was rated MODERATE quality due to smaller sample sizes. Implant stability received HIGH confidence due to precise, consistent measurements. Ridge dimensional changes were MODERATE quality due to measurement variability across studies. Safety evidence was MODERATE due to very low complication rates. Overall, evidence quality ranged from moderate to high, supporting strong clinical recommendations (Table 6).

Safety data were reassuring. Across 249 patients and 281 grafting sites, only three minor complications occurred in dentin groups (2.2%), and two in control groups (1.6%), so there was no significant difference. These complications consisted of transient swelling and minor wound infections—all resolved without treatment sequelae. Remarkably, no serious adverse events were observed, no immune reactions, no disease transmission, no graft failures, and no chronic inflammation. This suggests autogenous dentin grafts are exceptionally well-tolerated (Table 7).

## 4. Discussion

Null Hypothesis and Study Objective:

The null hypothesis posited that dentinal grafts would show no significant difference in bone regeneration, graft integration, or implant outcomes compared to conventional xenografts or natural socket healing. Our systematic analysis of eight randomized controlled trials provides robust evidence for rejection of this null hypothesis.

Key Findings Rejecting the Null Hypothesis:

1. New bone formation (primary outcome): Dentinal grafts produced significantly more new bone than xenografts (12.4% greater, 95% CI: 6.8–18.0%, *p* < 0.001), statistically rejecting the null hypothesis. This difference is both statistically significant and clinically meaningful, suggesting superior osteogenic potential of dentinal grafts.

2. Graft integration: The null hypothesis of equivalent residual material was rejected (8.6% less residual material with dentin grafts, *p* < 0.001), indicating faster and more complete graft integration compared to xenografts.

3. Implant success (non-inferiority): While implant stability was equivalent between groups (*p* = 0.51), dentinal grafts demonstrated non-inferiority to xenografts, failing to reject the equivalence hypothesis for this outcome but supporting the superiority hypothesis for bone formation and integration.

4. Safety profile: The null hypothesis of equivalent adverse events could not be rejected (*p* = 0.71), with both groups showing excellent safety profiles (only 2.2% minor complications in dentin group). This systematic review thus provides strong evidence that dentinal grafts offer meaningful clinical advantages over conventional xenografts for bone preservation after tooth extraction, while maintaining excellent implant outcomes and safety comparable to established materials.

This systematic analysis of eight randomized controlled trials provides convincing evidence that dentinal grafts offer meaningful clinical advantages for bone preservation after tooth extraction. The key findings merit emphasis: these materials produce 12.4% more new bone than standard xenografts (Table 4), integrate more completely with only 8.6% residual material remaining, achieve outcomes equivalent to xenografts regarding implant success (Table 5), and dramatically outperform natural healing by preventing 60–75% of expected bone loss [18,19,20,21,22,23,24,25]. Most remarkably, these results come with an exceptionally favorable safety profile and lower costs than commercial alternatives (Table 5).

The compositional similarities between dentine and bone facilitate seamless integration [9,10]. Dentin possesses a tubular microstructure that appears to promote cell migration and new blood vessel formation better than the larger pores in xenografts [7,9,26]. Importantly, dentin naturally contains growth-promoting substances such as bone morphogenetic proteins and transforming growth factor-beta [10] that stimulate bone cell activity [11]. These factors remain most bioavailable when partial demineralization is used with enough mineral removal to expose growth factors, but enough mineral retention to maintain structural integrity and provide stability [24,26]. This probably explains why partially demineralized and mineralized preparations outperformed completely demineralized material in the included trials [18,24,25].

Mechanically, dentin provides superior properties compared to cancellous bone grafts: roughly 30–40 times greater compressive strength and 20 times greater tensile strength [9,26]. This means the graft maintains its shape and volume during the critical early healing phase [13], preventing the graft collapse that sometimes occurs with weaker materials. The resorption pattern also appears optimal—faster than xenografts, which may persist for years, but slower than autogenous bone, creating a smooth transition as new bone gradually replaces graft material [8,18].

From a practical standpoint, dentinal grafts offer substantial advantages. Using the patient’s own extracted tooth eliminates any disease transmission risk, immunological concerns [7,8], or ethical objections. Processing typically requires only 15–30 min chairside using commercially available devices [9,10,14,26]. The absence of need for a second surgical site and cost-effectiveness make dentinal grafts particularly attractive for patients who value autogenous materials or face cost barriers [7,9].

Findings of this systematic review align with other recent evidence. A 2023 meta-analysis by Mahardawi et al. [26] reported comparable implant stability with dentin grafts, consistent with our ISQ findings. Gual-Vaqués (2018) [27] reported high implant success with tooth-derived materials, similar to our pooled rate. Sánchez-Labrador’s 2023 review concluded that dentin is effective for ridge preservation [28] supporting our main findings [26,27]. Furthermore, a recent systematic review by Ahamed et al. [29] evaluated various management techniques for peri-implant gaps and identified xenografts and alloplastic grafts as superior in preserving bone volume. This finding corroborates the importance of selecting appropriate biomaterials to maintain bone integrity around implants following placement, demonstrating that bone preservation strategies initiated at tooth extraction continue to influence long-term implant stability and osseointegration [29].

The review makes several contributions beyond previous systematic reviews. By focusing exclusively on randomized trials, higher-certainty evidence has been provided than reviews including case series [25,26]. Our meta-analysis directly pooled histomorphometric data from the included studies’ specific measurements of actual bone formation. The subgroup analyses identified that processing method matters substantially [18,24,25], with partially demineralized material showing optimal results. GRADE assessment provides transparent quality evaluation [16]. Our inclusion of recent (2024–2025) studies keeps the evidence current with the latest technological refinements.

However, significant limitations must be acknowledged. First, marked heterogeneity in dentin processing protocols substantially limited direct study comparability. The eight included trials employed four distinct dentin preparation methods: whole-tooth grafts (AWTGs), completely demineralized dentin matrices (DDMs), mineralized dentin matrices (MDMs), and partially demineralized dentin matrices (PDDMs), each with variable demineralization degrees and processing times. This heterogeneity (I^2^ values ranging from 28 to 42% for bone formation outcomes) reflects inconsistent clinical implementation and raises questions about the generalizability of findings. No standardized protocol exists for chairside dentin processing regarding demineralization duration (reported as 10–30 min across studies), particle size specifications, or final material characteristics. Consequently, while our subgroup analysis identified PDDM as most effective, practitioners cannot reliably replicate optimal preparation conditions without standardized guidelines.

Second, follow-up periods ranged from 4 to 18 months (median, 6 months), which is insufficient to assess long-term implant stability beyond two to three years, durability of bone maintenance, or potential delayed complications. Most studies did not assess outcomes beyond six months, thus limiting our ability to evaluate secondary bone resorption patterns or changes in peri-implant bone levels over extended periods. Only one study (Santos et al., 2021) [18] provided 18-month data, representing incomplete long-term assessment.

Third, only six of the eight studies (75%) provided complete histomorphometric data, limiting meta-analysis power and potentially introducing bias if studies with missing data differed systematically in their findings. The cohort size of 249 patients across 281 grafting sites, while adequate for demonstrating efficacy, remains modest for high-confidence recommendations in diverse clinical populations. Additionally, heterogeneous measurement methods (CBCT vs. clinical calipers) and inconsistent anatomical landmarks for ridge dimensional assessment introduce measurement variability not fully accounted for in statistical analyses.

Heterogeneity analysis: Heterogeneity in study design and processing methods. The heterogeneity identified in this analysis warrants detailed examination. While statistical heterogeneity was moderate for bone formation (I^2^ = 42%), the clinical heterogeneity—reflecting differences in participant populations, procedural variations, and measurement techniques—was substantial. Processing methods differed not only in regard to demineralization degree but also implementation: some studies used chairside processing devices (AutoBT and NanoBone), while others employed pre-processed materials. Demineralization times ranged from 10 min (some PDDM protocols) to 30 min (complete DDM protocols), directly influencing growth factor bioavailability and material strength. Particle size varied from 250 to 1000 μm across studies, affecting handling properties and resorption kinetics. These variations, though partially captured in our subgroup analyses by processing method (Figure 4), remain incompletely characterized and represent a major barrier to clinical implementation. The apparent superiority of PDDM and MDM over complete DDM (Figure 4: +18.0% vs. +5.2% new bone formation, *p* < 0.001) likely reflects optimal balance between growth factor exposure and structural integrity, yet the precise demineralization parameters achieving this balance remain undefined. Future standardization efforts must address these specific variables: (1) demineralization duration and method (chemical agents, concentrations, and temperature), (2) particle size ranges and size distribution, (3) sterilization protocols and their impact on material properties, (4) storage conditions and shelf-life effects on bioactivity, and (5) processing validation methods to ensure consistency. The lack of standardization extends to implant placement timing, use of adjunctive membranes, and socket anatomy classifications, which varied across studies and likely influenced outcomes independently of dentin graft efficacy. These confounding factors necessitate standardized protocols for future trials.

These limitations collectively reduce the certainty of evidence and highlight the urgent need for standardized protocols and long-term prospective studies. Important research gaps remain. Long-term studies tracking implant success and peri-implant health for 5–10 years would clarify durability [5,6]. Head-to-head comparisons with autogenous bone would establish whether dentin truly matches this gold standard [7,8]. Standardization studies could identify optimal processing parameters [13,26]. Mechanistic research elucidating growth factor release and cellular responses would deepen understanding, as shown by Grawish et al., Bessho et al., and Yeomans et al. [10,11,12]. Investigations in special populations (heavy smokers, diabetic patients, and elderly) would clarify applicability. Cost-effectiveness analyses would quantify economic benefits. Advanced applications, including vertical ridge augmentation, large defect reconstruction, and sinus lifting, need investigation [6,9].

Research priorities: Addressing the heterogeneity and follow-up gaps identified above should be paramount. Research priorities include the following:

(1) Standardization development: Consensus guidelines specifying precise processing parameters—demineralization duration, chemical agents and concentrations, particle size ranges, and sterilization methods—must be developed through multidisciplinary collaboration between clinicians, material scientists, and regulatory bodies. Comparative device studies evaluating different chairside processing systems (AutoBT, NanoBone, and others) should identify which platforms reproducibly achieve optimal material characteristics.

(2) Long-term outcome studies: Large prospective studies (minimum 100 patients per group) with 5–10-year follow-ups are essential to assess secondary bone resorption, long-term implant stability quotient (ISQ) changes, marginal bone loss kinetics, and late-stage complications. These studies should include periodic CBCT assessment at 6, 12, 24, 36, 60, and 120 months post-extraction.

(3) Standardized measurement protocols: Unified imaging protocols (CBCT slice thickness, orientation, and measurement landmarks) and outcome assessment tools must be adopted across research centers to enable meaningful meta-analyses and reduce measurement heterogeneity.

(4) Large multi-center randomized trials: Rigorous RCTs enrolling > 100 patients per group, with randomized assignment to specific standardized processing methods, and double-blinded outcome assessment, are needed to definitively establish which PDDM, MDM, or other preparations achieve superior long-term outcomes.

(5) Special populations: Studies in high-risk groups (heavy smokers, diabetic patients, immunocompromised individuals, and elderly > 75 years) should clarify whether dentin graft efficacy differs from general populations.

(6) Mechanistic research: Investigations elucidating growth factor release kinetics, cellular responses, vascularization patterns, and immune tolerance mechanisms will deepen our understanding of why PDDM/MDM outperforms DDM and inform future optimization.

(7) Cost-effectiveness analyses: Health economic studies comparing dentin grafting with xenografts and autogenous bone will quantify clinical and financial benefits across diverse healthcare systems.

Until these research priorities are systematically addressed, practitioners should view current recommendations as having moderate-to-high evidence quality but acknowledge the limitations imposed by heterogeneity and short follow-up periods [17,26,27,28,29].

Sticky tooth grafts with PRF/CGF as antibiotic carriers: Building on the superior bone regeneration demonstrated by conventional dentinal grafts in this review, future investigations should explore enhanced composite materials combining demineralized dentin with platelet-rich fibrin (PRF) or concentrated growth factors (CGFs)—termed “sticky tooth” grafts. Such bioactive scaffolds could synergistically combine the osteogenic potential and mechanical strength established in our meta-analysis with additional benefits of PRF/CGF.

Recent evidence shows that antibiotic-loaded PRF (AL-PRF) exhibits significant antibacterial activity against bacterial strains and outperforms alternative carriers such as collagen sponges with sustained antimicrobial release [30].

Integrating antibiotic-carrying PRF/CGF into dentinal grafts could provide dual benefits: enhanced bone regeneration (building on our findings of 12.4% greater new bone formation) and localized infection prevention particularly valuable in immunocompromised or complex extraction cases. The adhesive properties of PRF/CGF (“sticky”) would also improve clinical handling compared to conventional grafts, facilitating easier placement and retention in extraction sockets. While current evidence remains limited to in vitro studies, clinical trials investigating whether sticky tooth preparations offer additional benefits over conventional dentinal grafts are warranted. Given the excellent outcomes (12.4% greater bone formation, 8.6% less residual material, and 95% + implant success) and safety profile demonstrated in this review, exploring sticky tooth preparations represents a logical next-generation development in autogenous bone regeneration.

## 5. Conclusions

Dentinal grafts represent a safe and effective alternative to conventional xenografts for alveolar bone preservation. Our meta-analysis demonstrates superior new bone formation (12.4% greater), faster graft integration (8.6% less residual material), and equivalent implant success rates (>95%) with excellent safety (2.2% minor complications). Compared to natural healing, dentinal grafts reduce bone loss by 60–75%.

Clinically, dentinal grafts should be considered when patients prefer autogenous materials and suitable teeth are available. The recommendation is based on high-quality randomized trials. Future research should prioritize long-term studies with at least 5–10-year follow-ups, standardization of processing methods, and advanced applications. With sound scientific rationale and robust evidence, dentinal grafts are positioned to become standard care in implant dentistry.

## Figures and Tables

**Table 1:** Characteristics of included studies (meta-analysis). Characteristics of the eight randomized controlled trials (RCTs) included in the systematic review examining dentin grafts for alveolar ridge preservation and implant site development. Studies were conducted across multiple countries between 2017 and 2023, with sample sizes ranging from 13 to 52 participants. Study designs included parallel RCTs, prospective RCTs, and split-mouth designs.

Study ID	First Author, Year	Country	Study Design	Sample Size	Intervention	Control	Clinical Application	Follow-Up	Journal	DOI/Registry	Risk of Bias
1	Elfana et al., 2021 [17]	Egypt	RCT, Parallel	20 patients/20 sites	AWTG vs. ADDG	AWTG vs. ADDG (both dentin)	Alveolar ridge preservation	6 months	*Clin. Oral Implants Res.*	https://doi.org/10.1111/clr.13722	Low
2	Santos et al., 2021 [18]	Portugal	RCT, Parallel	52 patients/66 implants	MDM (mineralized dentin matrix)	Bio-Oss xenograft	Ridge preservation + delayed implant	18 months	*Clin. Oral Implants Res.*	https://doi.org/10.1111/clr.13765	Low
3	Li et al., 2018 [19]	China	RCT, Prospective	40 patients/45 implants	DDM (demineralized dentin matrix)	Bio-Oss xenograft	Immediate implant + GBR (periodontitis)	6 months	*Clin. Implants Dent. Relat. Res.*	https://doi.org/10.1111/cid.12674	Low
4	Pang et al., 2017 [20]	South Korea	RCT, Prospective	24 patients/24 sites	AutoBT (demineralized dentin)	Bio-Oss xenograft	Post-extraction bone augmentation	6 months	*Clin. Oral Implants Res.*	https://doi.org/10.1111/clr.12885	Low to moderate
5	Hussain et al., 2023 [21]	Iraq	RCT, Parallel	32 patients/32 sites	Autogenous dentin biomaterial	Natural socket healing (no graft)	Alveolar ridge preservation	4 months	*Biomed. Res. Int.*	TCTR20220615002	Low
6	Sapoznikov et al., 2023 [22]	Israel	RCT, Semi-double-blind	36 patients/36 sites	Porcine dentin-derived graft	Bone-derived xenograft	Alveolar ridge preservation	6 months	*Clin. Oral Implants Res.*	NCT03150472	Low
7	Kuperschlag et al., 2020 [23]	Israel	RCT, Split-mouth	13 patients/26 sites	ADG (autogenous dentin graft)	No graft (hemostatic sponge)	Third molar socket grafting	12 months	*Compend. Contin. Educ. Dent.*	https://doi.org/10.1563/aaid-joi-D-19-00213	Moderate
8	Yang et al., 2023 [24]	China	RCT, Parallel	32 patients/32 sites	APDDM (partially demineralized)	Spontaneous healing (No graft)	Socket preservation (periodontitis)	6 months	*Clin. Implant Dent. Relat. Res.*	https://doi.org/10.1111/cid.13247	Low to moderate

Footnotes: AWTG = autologous whole-tooth graft; ADDG = autogenous demineralized dentin graft. MDM = mineralized dentin matrix; DDM = demineralized dentin matrix. AutoBT = autogenous bone tissue; ADG = autologous dentin graft. APDDM = autogenous partially demineralized dentin matrix. GBR = guided bone regeneration; RCT = randomized controlled trial. Risk of bias: low = minimal concerns; low to moderate = some concerns identified; moderate = notable limitations. Study duration: Follow-up periods ranged from 4 to 18 months post-extraction/grafting. All studies were published between 2017 and 2023 in peer-reviewed journals.

**Table 2:** Subgroup analysis examining impact of dentin processing method on new bone formation outcomes. Data demonstrate that dentin preparation method significantly influences efficacy, with mineralized and partially demineralized preparations substantially outperforming completely demineralized material. Partially demineralized dentin showed the greatest advantage (+18.0%, 95% CI 11.2–24.8%, *p* < 0.001).

Processing Method	Mean Difference vs. Xenograft	95% CI	*p*-Value	Studies	Interpretation
Completely Demineralized Dentin (DDM)	+5.2%	(−2.1 to 12.5)	0.16	1–2	Not significant—smallest advantage
Mineralized Dentin (MDM)	+12.4%	(6.8 to 18.0)	<0.001	1–2	Significant—substantial advantage
Partially Demineralized (PDDM)	+18.0%	(11.2 to 24.8)	<0.001	1–2	Highest significant—greatest advantage

**Table 3:** Histomorphometric outcomes. Histomorphometric analysis results from included studies showing new bone formation percentage, residual graft material percentage, connective tissue area, and bone marrow area. Data demonstrate that dentin grafts produce significantly greater new bone formation (pooled MD, 12.4%; 95% CI, 6.8–18.0%; *p* < 0.001) compared to xenografts, with faster graft resorption indicating superior integration.

Study	Group Type	New Bone Formation (%)	Residual Graft (%)	Connective Tissue (%)	Bone Marrow Area	Follow-Up	*p*-Value	Key Finding
Elfana, 2021—AWTG [17]	Dentin graft	37.55 ± 8.94	17.05 ± 5.58	45.4 ± 4.06	Not reported	6 months	>0.05	Similar effectiveness; both prevent resorption
Elfana, 2021—ADDG [17]	Dentin graft	48.4 ± 11.56	11.45 ± 4.13	40.15 ± 7.73	Not reported	6 months	>0.05	Better graft remodeling and integration
Santos 2021—MDM [18]	Dentin graft	47.3 (mean)	12.2	Not reported	Not reported	6 months	<0.001	Higher new bone, lower residual graft
Santos, 2021—Bio-Oss [18]	Xenograft	34.9 (mean)	22.1	Not reported	Not reported	6 months	<0.001	Lower new bone, higher residual graft
Pang, 2017—AutoBT [20]	Dentin graft	31.24 ± 10.5	Not reported	Not reported	Not reported	6 months	>0.05 (NS)	Comparable to Bio-Oss
Pang, 2017—Bio-Oss [20]	Xenograft	35.0 ± 10.5	Not reported	Not reported	Not reported	6 months	>0.05 (NS)	Comparable to AutoBT
Hussain, 2023—ADB [21]	Dentin graft	Trabecular: 1.33 ± 0.24 mm^2^	Not reported	Not reported	0.49 ± 0.19 mm^2^	4 months	<0.001	Significantly higher trabecular bone
Hussain, 2023—control [21]	Natural healing	Trabecular: 0.72 ± 0.23 mm^2^	NS	Not reported	1.12 ± 0.23 mm^2^	4 months	<0.001	Higher bone marrow, thin trabeculae
Sapoznikov, 2023—porcine dentin [22]	Dentin xenograft	60.75	Not reported	Not reported	Not reported	4 months	0.0084	Significantly more new bone formation
Sapoznikov, 2023—porcine bone [22]	Bone xenograft	42.81	Not reported	Not reported	Not reported	4 months	0.0084	Less new bone, lower radiodensity
Kuperschlag, 2020—ADG [23]	Dentin graft	72.55 ± 12.14	10.61 ± 5.37	16.84 ± 9.18	Not reported	4 months	0.613	Similar to xenograft combination
Yang, 2023—BX + AB [24]	Xenograft + bone	69.61 ± 13.53	12.31 ± 7.83	18.07 ± 6.93	Not reported	4 months	0.613	Similar to ADG alone

Abbreviations: AWTG = autologous whole-tooth graft, ADDG = autogenous demineralized dentin graft, MDM = mineralized dentin matrix, AutoBT = autogenous bone tissue, ADB = autogenous dentin bone, ADG = autologous dentin graft, BX = Bone Extract, AB = autogenous bone, NS = not significant.

**Table 4:** Ridge dimensional changes. Linear measurements of horizontal and vertical alveolar ridge dimensional changes following tooth extraction and grafting procedures. Dentin grafts demonstrated significant benefit in reducing ridge resorption compared to natural healing controls (60–75% less bone loss, *p* < 0.001). Changes measured from baseline (immediately post-extraction) to follow-up (4–6 months).

Study	Group	Horizontal Change (mm)	Vertical Change—Buccal (mm)	Vertical Change—Lingual (mm)	Measurement Method	Follow-Up	Statistical Significance	Clinical Outcome
Elfana, 2021 [17]	AWTG	−0.85 ± 0.38	−0.61 ± 0.20	−0.66 ± 0.31	CBCT	6 months	*p* > 0.05 (between AWTG and ADDG)	Minimal bone loss; adequate for implant
Elfana, 2021 [17]	ADDG	−1.02 ± 0.45	−0.72 ± 0.27	−0.56 ± 0.24	CBCT	6 months	*p* > 0.05 (between AWTG and ADDG)	Minimal bone loss; adequate for implant
Hussain, 2023 [21]	ADB (dentin)	Significantly less reduction *	Significantly less reduction *	Not separately measured	CBCT	4 months	*p* < 0.001 (ADB vs. Control)	60–70% less horizontal loss vs. control
Hussain, 2023 [21]	Control (natural healing)	Marked reduction *	Marked reduction *	Not separately measured	CBCT	4 months	*p* < 0.001 (ADB vs. Control)	Severe horizontal and vertical resorption
Yang, 2023 [24]	APDDM (dentin)	−1.2 ± 0.8	−0.9 ± 0.6	Not separately measured	CBCT	6 months	*p* < 0.05 (APDDM vs. Control)	65–75% less bone loss vs. control
Yang, 2023 [24]	Control (natural healing)	−3.5 ± 1.2	−2.8 ± 1.1	Not separately measured	CBCT	6 months	*p* < 0.05 (APDDM vs. Control)	Significant alveolar collapse
Santos, 2021 [18]	MDM (dentin)	Not reported	Not reported	Not reported	Clinical (calipers)	6 months	Adequate preservation in both groups	Successful delayed implant placement
Santos, 2021 [18]	Bio-Oss (xenograft)	Not reported	Not reported	Not reported	Clinical (calipers)	6 months	Adequate preservation in both groups	Successful delayed implant placement

Abbreviations: AWTG = autologous whole-tooth graft; ADDG = autogenous demineralized dentin graft; ADB = autogenous dentin biomaterial; APDDM = autogenous partially demineralized dentin matrix; MDM = mineralized dentin matrix; Bio-Oss = bovine xenograft; CBCT = cone beam computed tomography; mm = millimeters; vs. = versus. *—Qualitative descriptors like significant reduction or marked reduction.

**Table 5:** Implant stability outcomes (ISQs)—primary ISQ measurements. Implant stability outcomes measured by implant stability quotient (ISQ) from the three studies (*n* = 3 studies, 138 participants) that reported primary and secondary ISQ data for meta-analysis. Data demonstrate non-inferiority of dentin grafts compared to xenografts, with comparable ISQ values, excellent implant success rates (95–100%), and minimal marginal bone loss. Meta-analysis pooled result: MD −0.8 (95% CI −3.2 to 1.6, *p* = 0.51).

Study	Group	Primary ISQ (at Placement)	Secondary ISQ (2+ Months)	Implant Success Rate	Marginal Bone Loss (mm)	Follow-Up	Statistical Comparison	Key Findings
Santos, 2021 [18]	MDM (dentin)	77.1 ± 6.9	81.8 ± 5.1 (at 2 months)	100% (26/26)	0.12 ± 0.08 (at 18 months)	18 months	*p* > 0.05 (no difference)	Excellent osseointegration, similar to xenograft
Santos, 2021 [18]	Bio-Oss (xenograft)	77.0 ± 5.9	80.1 ± 3.8 (at 2 months)	100% (26/26)	0.15 ± 0.11 (at 18 months)	18 months	*p* > 0.05 (no difference)	Excellent osseointegration
Li, 2018 [19]	DDM (dentin)	Similar to Bio-Oss	Similar to Bio-Oss	95.6% (43/45)	Similar between groups	18 months	*p* > 0.05 (no difference)	Comparable stability in periodontitis cases
Li, 2018 [19]	Bio-Oss (xenograft)	Similar to Bio-Oss	Similar to Bio-Oss	95.6% (43/45)	Similar between groups	18 months	*p* > 0.05 (no difference)	Comparable stability
Pang, 2017 [20]	AutoBT (Dentin)	72.80 ± 10.81	Not measured	100% (15/15)	Not reported	6 months	*p* > 0.05 (no difference)	Adequate primary stability
Pang, 2017 [20]	Bio-Oss (Xenograft)	70.0 ± 12.86	Not measured	100% (9/9)	Not reported	6 months	*p* > 0.05 (no difference)	Adequate primary stability

Abbreviations: ISQ = implant stability quotient; MDM = mineralized dentin matrix; DDM = demineralized dentin matrix; AutoBT = autogenous bone tissue; mm = millimeters; *p* = *p*-value.

**Table 6:** GRADE evidence profile. Summary of findings and certainty of evidence assessment. GRADE (Grading of Recommendations Assessment, Development, and Evaluation) evidence profile assessing the certainty of evidence for all primary outcomes using standardized methodology. Overall certainty ranges from HIGH (new bone formation, implant stability, and implant success) to MODERATE (residual graft material, ridge dimensional changes, and complications). Assessment evaluates risk of bias, inconsistency, indirectness, imprecision, publication bias, and large effects.

Outcome	Number of Studies	Number of Participants	Effect Estimate (95% CI)	*p*-Value	Study Design	Risk of Bias	Inconsistency (I^2^)	Indirectness	Imprecision	Publication Bias	Large Effect	Certainty of Evidence	Interpretation
New bone formation (%) vs. xenografts	4	172	MD 12.4% (6.8 to 18.0%)	<0.001	RCTs	Not serious (−0)	Not serious (I^2^ = 42%) (−0)	Not serious (−0)	Not serious (−0)	Undetected (−0)	Yes (+1)	⊕⊕⊕⊕ HIGH	Dentin grafts produce significantly more new bone
Residual graft material (%) vs. xenografts	3	109	MD −8.6% (−11.2 to −6.0%)	<0.001	RCTs	Not serious (−0)	Not serious (I^2^ = 28%) (−0)	Not serious (−0)	Serious (small sample) (−1)	Undetected (−0)	No (+0)	⊕⊕⊕◯ MODERATE	Dentin grafts show faster resorption/better integration
Primary implant stability (ISQ)	3	138	MD −0.8 (−3.2 to 1.6)	0.51	RCTs	Not serious (−0)	Not serious (I^2^ = 0%) (−0)	Not serious (−0)	Not serious (−0)	Undetected (−0)	No (+0)	⊕⊕⊕⊕ HIGH	Dentin grafts are non-inferior to xenografts
Ridge dimensional changes vs. natural healing	2	64	Narrative synthesis: significant benefit *	<0.05	RCTs	Not serious (−0)	Serious (different methods) (−1)	Not serious (−0)	Serious (small sample) (−1)	Undetected (−0)	Yes (+1)	⊕⊕⊕◯ MODERATE	Dentin grafts significantly reduce bone loss vs. control
Implant success rate	4	212	96.4% vs. 94.2%	>0.05	RCTs	Not serious (−0)	Not serious (−0)	Not serious (−0)	Not serious (−0)	Undetected (−0)	No (+0)	⊕⊕⊕⊕ HIGH	Excellent implant survival with dentin grafts
Complications and adverse events	8	249	RR 1.37 (0.26 to 7.21)	0.71	RCTs	Not serious (−0)	Not serious (−0)	Not serious (−0)	Serious (wide CI, few events) (−1)	Undetected (−0)	No (+0)	⊕⊕⊕◯ MODERATE	Dentin grafts are safe with minimal complications

Abbreviations: GRADE = Grading of Recommendations Assessment, Development, and Evaluation; MD = mean difference; RR = relative risk; CI = confidence interval; I^2^ = heterogeneity statistic; ISQ = implant stability quotient; RCTs = randomized controlled trials. Symbols: I^2^ = I-squared statistic (measure of heterogeneity/inconsistency); % = percentage; vs. = versus; *n* = number. ⊕⊕⊕⊕ = HIGH certainty: Further research very unlikely to change confidence in effect estimate, ⊕⊕⊕◯ = MODERATE certainty: Further research may change confidence in effect estimate. * = *p*-value < 0.05.

**Table 7:** Complication and adverse events. Safety profile and adverse event documentation from all eight included studies (*n* = 249 participants, 262 sites). Comprehensive assessment across 4–18-month follow-up periods demonstrates excellent safety with minimal complications (1.6–2.2%). No graft failures, immunological reactions, or disease transmission events were reported, confirming dentin grafts are safe for clinical implementation.

Study	Total Participants	Dentin Group (*n*)	Control Group (*n*)	Complications in Dentin Group	Complications in Control Group	Type of Complications	Graft Failures	Immunological Reactions	Disease Transmission	Follow-Up	Overall Safety Assessment
Elfana, 2021 [17]	20	10 + 10 (both dentin)	N/A (both dentin)	None (0/20)	None (0/20)	No complications reported	N/A	N/A	N/A	6 months	Excellent
Santos, 2021 [18]	52	26	26	None (0/26)	None (0/26)	Uneventful healing in all cases	N/A	N/A	N/A	18 months	Excellent
Li, 2018 [19]	40	22	18	Wound infection: 1/22	Wound infection: 1/18	Minor wound infection, resolved with antibiotics	N/A	N/A	N/A	18 months	Excellent
Pang, 2017 [20]	24	15	9	None (0/15)	None (0/9)	Uneventful healing	N/A	N/A	N/A	6 months	Excellent
Hussain, 2023 [21]	32	16	16	None (0/16)	None (0/16)	No complications; excellent wound healing	N/A	N/A	N/A	4 months	Excellent
Sapoznikov, 2023 [22]	36	20	16	Minor swelling: 2/20	Minor swelling: 1/16	Transient swelling (days 1–3), resolved spontaneously	N/A	N/A	N/A	6 months	Excellent
Kuperschlag, 2020 [23]	13	13	13	None (0/13)	None (0/13)	Uneventful healing; no periodontal complications	N/A	N/A	N/A	12 months	Excellent
Yang, 2023 [24]	32	16	16	None (0/16)	None (0/16)	No complications reported	N/A	N/A	N/A	6 months	Excellent
Pooled total	249	138	124	3/138 (2.2%)	2/124 (1.6%)	All minor; no serious adverse events	0/249 (0%)	0/249 (0%)	0/249 (0%)	4–18 months	EXCELLENT—Safe for clinical use

Abbreviations: *n* = number of participants; N/A = not applicable.

## Data Availability

No new data were created or analyzed in this study. Supplementary data are available by contacting the first author. Supplementary Table S1 for sensitivity analysis; Supplementary Table S2 for subgroup analysis processing; Supplementary Table S3 with PRISMA Checklist; Supplementary Table S4 with search documentation; Supplementary Table S5 with excluded studies; Supplementary Table S6 with excluded studies’ characteristics.

## Supplementary Materials

The following supporting information can be downloaded at https://www.mdpi.com/article/10.3390/dj14020100/s1, Supplementary Table S1: Sensitivity Analysis Results; Supplementary Table S2: Subgroup Analysis: Processing Method; Supplementary Table S3: PRISMA 2020 Checklist; Supplementary Table S4: Search Strategy Documentation; Supplementary Table S5: Excluded Studies Summary; Supplementary Table S6: Characteristics of Excluded Studies (After Full-Text Review).
