# Supplementary material for: Dentinal Grafts, a Promising Material for Alveolar Defects: A Systematic Review and Meta-Analysis
Source: Dent J (Basel). 2026 Feb 10;14(2):100. doi: 10.3390/dj14020100 (PMC12940014; doi:10.3390/dj14020100)
Supplement: Supplementary file 1 [file dentistry-14-00100-s001.zip › Supplementary_Table_S1_Sensitivity_Analysis.pdf]

Supplementary Table 1: Sensitivity Analysis Results

Outcome	N Studies	N Participants	Effect Estimate (95% CI)	P-value	I² (%)	Conclusion
**New bone formation vs xenografts**	4	172	MD 12.4% (6.8-18.0%)	<0.001	42	Significant benefit
**New bone formation vs xenografts***	3	149	MD 14.2% (7.5-20.9%)	<0.001	38	Findings robust, increased effect
**Residual graft material vs xenografts**	4	109	MD -8.6% (-11.2 to -6.0%)	<0.001	28	Significant benefit
**Residual graft material vs xenografts***	3	92	MD -9.1% (-12.3 to -5.9%)	<0.001	22	Findings robust
**Primary implant stability (ISQ)**	3	138	MD -0.8 (-3.2 to 1.6)	0.51	0	Non-inferiority confirmed
**Primary implant stability (ISQ)***	3	122	MD -0.5 (-3.1 to 2.1)	0.68	0	Findings robust
**Implant success rate**	4	212	96.4% vs 94.2%	>0.05	60	No significant difference
**Implant success rate***	4	212	96.8% vs 94.5%	>0.05	75	Findings robust
**Ridge dimensional changes vs natural healing**	2	64	MD -2.3 mm (horizontal)	<0.05	N/A	Significant benefit
**Ridge dimensional changes vs natural healing***	2	64	Narrative synthesis	N/A	N/A	60-75% consistent with full analysis
**Complications and adverse events**	8	249	RR 1.37 (0.26-7.21)	0.71	N/A	Excellent safety
**Complications and adverse events***	8	249	RR 1.15 (0.15-8.82)	0.89	N/A	Findings robust, excellent safety

Table Caption: Sensitivity analysis results comparing full meta-analysis (all 8 studies) with restricted analysis (6 high-quality studies only), excluding Pang et al. (2017) for allocation concealment concerns and Yang et al. (2023) for missing data. Sensitivity analysis demonstrates robustness of primary findings with consistent effect directions and no qualitative changes in conclusions.

Footnotes:

- * Sensitivity analysis excluding Pang et al. (2017) due to allocation concealment concerns and Yang et al. (2023) due to missing histomorphometric data
- Full analysis: All 8 included studies; Sensitivity analysis: 6 high-quality studies only
- CI = Confidence Interval; MD = Mean Difference; RR = Risk Ratio
- ISQ = Implant Stability Quotient; N/A = Not Applicable

- I^2 = measure of statistical heterogeneity
- Results demonstrate that primary findings remain robust when excluding two lower-quality studies
- For new bone formation, effect estimates actually increased in sensitivity analysis (12.4% to 14.2%), indicating lower-quality studies were not driving results
- Sensitivity analysis confirms findings are not biased by quality of included studies
- Non-inferiority findings confirmed: dentin grafts perform equivalently to or better than xenografts
