# Supplementary material for: Dentinal Grafts, a Promising Material for Alveolar Defects: A Systematic Review and Meta-Analysis
Source: Dent J (Basel). 2026 Feb 10;14(2):100. doi: 10.3390/dj14020100 (PMC12940014; doi:10.3390/dj14020100)
Supplement: Supplementary file 1 [file dentistry-14-00100-s001.zip › Supplementary_Table_S2_Subgroup_Analysis_Processing.pdf]

Supplementary Table 2: Subgroup Analysis: Processing Method

Processing Method	Mean Difference vs Xenograft	95% CI	P-value	Studies	Interpretation
**Completely Demineralized Dentin (DDM)**	+5.2%	(-2.1 to 12.5)	0.16	1-2	Not significant - smallest advantage
**Mineralized Dentin (MDM)**	+12.4%	(6.8 to 18.0)	<0.001	1-2	Significant - substantial advantage
**Partially Demineralized (PDDM)**	+18.0%	(11.2 to 24.8)	<0.001	1-2	Highest significant - greatest advantage

Table Caption: Subgroup analysis examining impact of dentin processing method on new bone formation outcomes. Data demonstrate that dentin preparation method significantly influences efficacy, with mineralized and partially demineralized preparations substantially outperforming completely demineralized material. Partially demineralized dentin showed the greatest advantage (+18.0%, 95% CI 11.2-24.8%, $p<0.001$).

Footnotes:

- DDM = Demineralized Dentin Matrix (all mineral removed)
- MDM = Mineralized Dentin Matrix (mineral preserved)
- PDDM = Partially Demineralized Dentin Matrix (partial mineral removal)
- Subgroup analysis focused on new bone formation outcomes
- Processing method significantly influenced results ($p<0.05$)
- Partially demineralized dentin showed superior new bone formation compared to completely demineralized material
- Mineralized dentin preparations outperformed completely demineralized material
- Completely demineralized material showed smallest advantage over xenografts (+5.2%, not statistically significant)
- This suggests that retaining mineral content improves osteogenic potential
- Clinical implication: processing method selection influences clinical outcomes
