# Supplementary material for: Dentinal Grafts, a Promising Material for Alveolar Defects: A Systematic Review and Meta-Analysis
Source: Dent J (Basel). 2026 Feb 10;14(2):100. doi: 10.3390/dj14020100 (PMC12940014; doi:10.3390/dj14020100)
Supplement: Supplementary file 1 [file dentistry-14-00100-s001.zip › Supplementary_Table_S3_PRISMA_Checklist.pdf]

Supplementary Table 3: PRISMA 2020 Checklist

PRISMA Section	Completed (Yes/No)	Page/Location
**Title and Abstract**	Yes	Cover Page, Abstract
**Introduction - Rationale and Objectives**	Yes	Introduction, Methods
**Methods - Eligibility Criteria**	Yes	Methods - Eligibility section
**Methods - Information Sources**	Yes	Methods - Information Sources
**Methods - Search Strategy**	Yes	Methods - Search Strategy appendix
**Methods - Study Selection Process**	Yes	Methods - Study Selection section
**Methods - Data Extraction**	Yes	Methods - Data Extraction section
**Methods - Risk of Bias Assessment**	Yes	Methods - Risk of Bias section
**Methods - Effect Measures**	Yes	Methods - Effect Measures section
**Methods - Synthesis Methods**	Yes	Methods - Statistical Analysis section
**Methods - Reporting Bias Assessment**	Yes	Methods - Publication Bias section
**Methods - Certainty Assessment**	Yes	Methods - GRADE Assessment section
**Results - Study Selection**	Yes	Results - Study Selection (Figure 1)
**Results - Study Characteristics**	Yes	Results - Table 1 (Study Characteristics)
**Results - Risk of Bias in Studies**	Yes	Results - Figure 2, Risk of Bias analysis
**Results - Results of Individual Studies**	Yes	Results - Tables 3-6 (Outcome data)
**Results - Results of Meta-analyses**	Yes	Results - Figures 3-6 (Meta-analyses)
**Results - Reporting Biases**	Yes	Results - Reporting Bias discussion
**Results - Certainty of Evidence**	Yes	Results - Table 7 (GRADE profile)
**Discussion - Interpretation**	Yes	Discussion - Interpretation section
**Discussion - Implications**	Yes	Discussion - Implications for practice
**Discussion - Limitations**	Yes	Discussion - Limitations acknowledged
**Discussion - Conclusions**	Yes	Discussion - Conclusions stated
**Other - Registration and Protocol**	Yes	Protocol registration: PROSPERO registered
**Other - Data Availability**	Yes	Data availability: Available from corresponding author
**Other - Funding and Conflicts**	Yes	Funding and conflicts: Declared in manuscript

Table Caption: PRISMA 2020 Checklist verification documenting completed items and corresponding locations in the manuscript. All 26 mandatory items from the PRISMA 2020 guideline were completed and reported, demonstrating full compliance with international standards for transparent and comprehensive reporting of systematic reviews and meta-analyses.

Footnotes:

- PRISMA = Preferred Reporting Items for Systematic Reviews and Meta-Analyses
- Checklist version: PRISMA 2020 (updated guideline for reporting systematic reviews and meta-analyses)
- All 26 items from PRISMA 2020 checklist completed and reported
- This systematic review adheres to international standards for transparent reporting
- Complete PRISMA checklist available in supplementary materials
- Compliance with PRISMA guidelines ensures reproducibility and reduces reporting bias
- PROSPERO registration: All systematic reviews prospectively registered prior to study identification
- See PRISMA 2020 reference: Page MJ, et al. BMJ 2021;372:n71
