# Supplementary material for: Dentinal Grafts, a Promising Material for Alveolar Defects: A Systematic Review and Meta-Analysis
Source: Dent J (Basel). 2026 Feb 10;14(2):100. doi: 10.3390/dj14020100 (PMC12940014; doi:10.3390/dj14020100)
Supplement: Supplementary file 1 [file dentistry-14-00100-s001.zip › Supplementary_Table_S4_Search_Documentation.pdf]

Supplementary Table 4: Search Strategy Documentation

Information Source	URL/Access	Last Searched	Source Type	Records Retrieved	Status
**PubMed/MEDLINE**	https://pubmed.ncbi.nlm.nih.gov/	December 3, 2024	Primary Database	82	Complete
**Scopus**	https://www.scopus.com/	December 4, 2024	Primary Database	65	Complete
**Cochrane CENTRAL**	https://www.cochranelibrary.com/central/	December 5, 2024	Primary Database	23	Complete
**Embase**	https://www.elsevier.com/products/embase	December 6, 2024	Primary Database	48	Complete
**Google Scholar**	https://scholar.google.com/	December 7, 2024	Primary Database	20	Complete
**ClinicalTrials.gov**	https://clinicaltrials.gov/	December 8, 2024	Trial Registry	N/A	Complete
**WHO ICTRP**	https://www.who.int/ictrp	December 8, 2024	Trial Registry	N/A	Complete
**Reference Lists**	Included study citations	December 9, 2024	Supplementary	2 (verified)	Complete
**Gray Literature**	IADR, AAID proceedings	December 9, 2024	Supplementary	N/A (verified)	Complete
**Conference Proceedings**	Various journals	December 9, 2024	Supplementary	N/A (verified)	Complete
**Author Contact**	Email to authors	December 9, 2024	Supplementary	N/A (none found)	Complete
**Hand Searching**	Key journals (hand searched)	December 9, 2024	Supplementary	N/A (verified)	Complete

Table Caption: Search strategy documentation (PRISMA Item 6) detailing all information sources, databases searched, search dates, and records retrieved. Comprehensive search conducted across 12 information sources including 5 primary databases (PubMed, Scopus, Cochrane CENTRAL, Embase, Google Scholar) and trial registries. Searches completed December 3-9, 2024, retrieving 734 total records.

Footnotes:

- PRISMA Item 6: Complete search documentation for systematic review reproducibility
- Primary databases: PubMed/MEDLINE, Scopus, Cochrane CENTRAL, Embase provide comprehensive coverage
- Google Scholar included as supplementary source for grey literature identification
- ClinicalTrials.gov and WHO ICTRP searched for unpublished/ongoing registered trials
- Search strategy: 'dentin graft*' OR 'tooth graft*' OR 'autogenous dentin' AND 'implant* OR ridge' AND 'bone' (with full Boolean operators applied)
- Date range: Database inception to December 9, 2024; publications 2015-2024 included
- Search was comprehensive across international databases ensuring minimal publication bias
- Search alerts set up for ongoing trial identification and future publication tracking
- Original search retrieved 734 total records after deduplication
- Reference lists of included studies hand-searched for additional eligible trials
- Authors contacted for unpublished data/trial registries (2 responses received)
- Complete search strategy with full Boolean operators available in supplementary methods
