# Supplementary material for: Dentinal Grafts, a Promising Material for Alveolar Defects: A Systematic Review and Meta-Analysis
Source: Dent J (Basel). 2026 Feb 10;14(2):100. doi: 10.3390/dj14020100 (PMC12940014; doi:10.3390/dj14020100)
Supplement: Supplementary file 1 [file dentistry-14-00100-s001.zip › Supplementary_Table_S5_Excluded_Studies.pdf]

Supplementary Table 5: Excluded Studies Summary

Exclusion Category	Reason for Exclusion	Number of Studies	Representative Examples
**Not RCT (n=14)**	Observational studies, case series, prospective cohorts lacked randomization	14	Kim et al. 2020, Lee et al. 2019, Zhang et al. 2021
**Published before 2015 (n=8)**	Publications from 2010-2014 outside date range cutoff	8	Nampo et al. 2010, Murata et al. 2011, Al-Asfour et al. 2013
**Systematic reviews (n=7)**	Literature reviews, meta-analyses of existing RCTs	7	Mahardawi et al. 2023, Schwarz et al. 2019, Joshi et al. 2021
**Non-English language (n=3)**	Publications in Chinese, Italian, German without English translation	3	Schmidt et al. 2021 (German), Chen et al. 2022 (Chinese)
**Unpublished materials (n=2)**	Conference abstracts only, ongoing trials without published results	2	Brown et al. 2022 (ongoing), Johnson et al. 2023 (abstract)
**No comparison group (n=2)**	Single-arm studies, descriptive studies without control groups	2	Taylor et al. 2021 (case series), Wilson et al. 2020 (descriptive)

Table Caption: Summary of study exclusion categories and reasons after full-text review. Of 734 initial database records, 36 studies underwent full-text review and were excluded based on pre-defined PICOS eligibility criteria. Primary reasons for exclusion: non-RCT design (n=14), pre-2015 publication (n=8), systematic reviews (n=7), non-English language (n=3), unpublished data (n=2), and lack of comparison groups (n=2).

Footnotes:

- Total excluded after full-text review: 36 studies
- Studies excluded at title/abstract stage (n=125): non-dental topics, animal studies, in vitro studies, review articles
- Total database records screened: 734 (after deduplication from 1,024 original records)
- Inclusion criteria: Randomized Controlled Trial (RCT), peer-reviewed publication, English language, published 2015-2024, human studies
- PICOS criteria: Participants (humans requiring alveolar ridge preservation or implant site development), Interventions (dentin-based grafts)
- Comparators (xenografts, natural healing, autogenous bone, synthetic), Outcomes (new bone formation, implant stability, success)
- Study designs (RCTs with prospective or randomized allocation)
- Common reasons for exclusion: non-randomized designs, inadequate follow-up (<4 months), missing outcome data, historical controls
- Final included studies: 8 RCTs (n=249 participants, 262 sites)
- Excluded studies available for review upon request from corresponding author
