# Supplementary material for: Dentinal Grafts, a Promising Material for Alveolar Defects: A Systematic Review and Meta-Analysis
Source: Dent J (Basel). 2026 Feb 10;14(2):100. doi: 10.3390/dj14020100 (PMC12940014; doi:10.3390/dj14020100)
Supplement: Supplementary file 1 [file dentistry-14-00100-s001.zip › Supplementary_Table_S6_Excluded_Studies_Characteristics.pdf]

Table 2: Characteristics of Excluded Studies (After Full-Text Review)

Study ID	Author(s), Year	Study Design	Primary Reason for Exclusion	Specific Notes
1	Kim et al., 2020	Prospective cohort	Not RCT	Lacked randomization
2	Lee et al., 2019	Retrospective cohort	Not RCT	No random allocation
3	Zhang et al., 2021	Case series	Not RCT	Non-comparative design
4	Morales et al., 2022	Prospective case series	Not RCT	No control group
5	Wang et al., 2020	Retrospective analysis	Not RCT	Non-randomized observational
6	Nakamura et al., 2019	Prospective case series	Not RCT	No randomization
7	Gomez et al., 2018	Pilot study	Not RCT	Small non-randomized sample
8	Silva et al., 2021	Prospective study	Not RCT	Non-randomized observational
9	Martinez et al., 2020	Clinical study	Not RCT	No control group
10	Hernandez et al., 2022	Case-control	Not RCT	Non-randomized design
11	Park et al., 2021	Prospective cohort	Not RCT	No random allocation
12	Tanaka et al., 2019	Clinical series	Not RCT	Non-comparative
13	Costa et al., 2020	Retrospective review	Not RCT	Historical controls
14	Suzuki et al., 2023	Observational	Not RCT	No randomization
15	Binderman et al., 2014	RCT	Published before 2015	Excluded by date cutoff
16	Gomes et al., 2013	Clinical trial	Published before 2015	Pre-2015 publication
17	Jun et al., 2012	RCT	Published before 2015	Outside date range
18	Murata et al., 2011	Clinical study	Published before 2015	Pre-2015 publication
19	Nampo et al., 2010	Clinical trial	Published before 2015	Outside inclusion period
20	Al-Asfour et al., 2013	Experimental study	Published before 2015	Pre-2015 publication
21	Kabir et al., 2011	Clinical research	Published before 2015	Published before 2015
22	Grawish et al., 2014	Clinical trial	Published before 2015	Outside date range
23	Mahardawi et al., 2023	Systematic review	Published before 2015	Pre-2015 publication
24	Gual-Vaqus et al., 2018	Systematic review	Published before 2015	Not original research - Review of RCTs
25	Sánchez-Labrador et al., 2023	Systematic review	Published before 2015	Review article
26	Minetti et al., 2020	Review article	Not original research	Narrative review
27	Schwarz et al., 2019	Systematic review	Not original research	Meta-analysis
28	Joshi et al., 2021	Review article	Not original research	Literature review
29	Fernandez et al., 2022	Systematic review	Not original research	Review of existing studies
30	Chen et al., 2022	RCT	Non-English language	Published in Chinese
31	Rossi et al., 2020	Clinical trial	Non-English language	Published in Italian
32	Schmidt et al., 2021	RCT	Non-English language	Published in German
33	Johnson et al., 2023	RCT protocol	Unpublished data	Conference abstract only
34	Brown et al., 2022	Ongoing trial	Unpublished data	Trial registered but not completed
35	Taylor et al., 2021	Case series	No comparison group	Single-arm study
36	Wilson et al., 2020	Clinical study	No comparison group	Descriptive study only

Table Caption: Studies excluded after full-text review (n=36) with primary reasons for exclusion. All studies were assessed against pre-defined PICOS (Participants, Interventions, Comparators, Outcomes, Study design) eligibility criteria. Primary exclusion reasons: non-RCT study design (n=14), publications

predating 2015 (n=8), systematic reviews (n=7), non-English language (n=3), unpublished materials (n=2), and studies lacking appropriate comparison groups (n=2).

Footnotes:

- RCT = Randomized Controlled Trial
- Total studies examined during full-text review: 36
- Studies excluded from systematic review based on pre-defined PICOS eligibility criteria
- Not RCT (n=14): Observational cohorts, case series, and prospective studies without randomization
- Published before 2015 (n=8): Publications outside the defined date range cutoff (2015-2024)
- Not original research (n=7): Systematic reviews, literature reviews, narrative reviews, meta-analyses
- Non-English language (n=3): Publications in Chinese, Italian, or German without English translation available
- Unpublished data (n=2): Conference abstracts and ongoing trials without published results
- No comparison group (n=2): Single-arm studies and descriptive studies lacking appropriate controls
- PICOS criteria: Participants (humans), Interventions (dentin grafts), Comparators (xenografts/controls), Outcomes (bone/implant), Study design (RCTs)
- Complete details and reasons for each excluded study available from corresponding author upon request
